# Supplementary material for: Structural Features of DNA in tRNA Genes and Their Upstream Sequences
Source: Int J Mol Sci. 2024 Nov 1;25(21):11758. doi: 10.3390/ijms252111758 (PMC11547032; doi:10.3390/ijms252111758)
Supplement: Supplementary file 1 [file ijms-25-11758-s001.zip › ijms-3060079-supplementary.pdf]

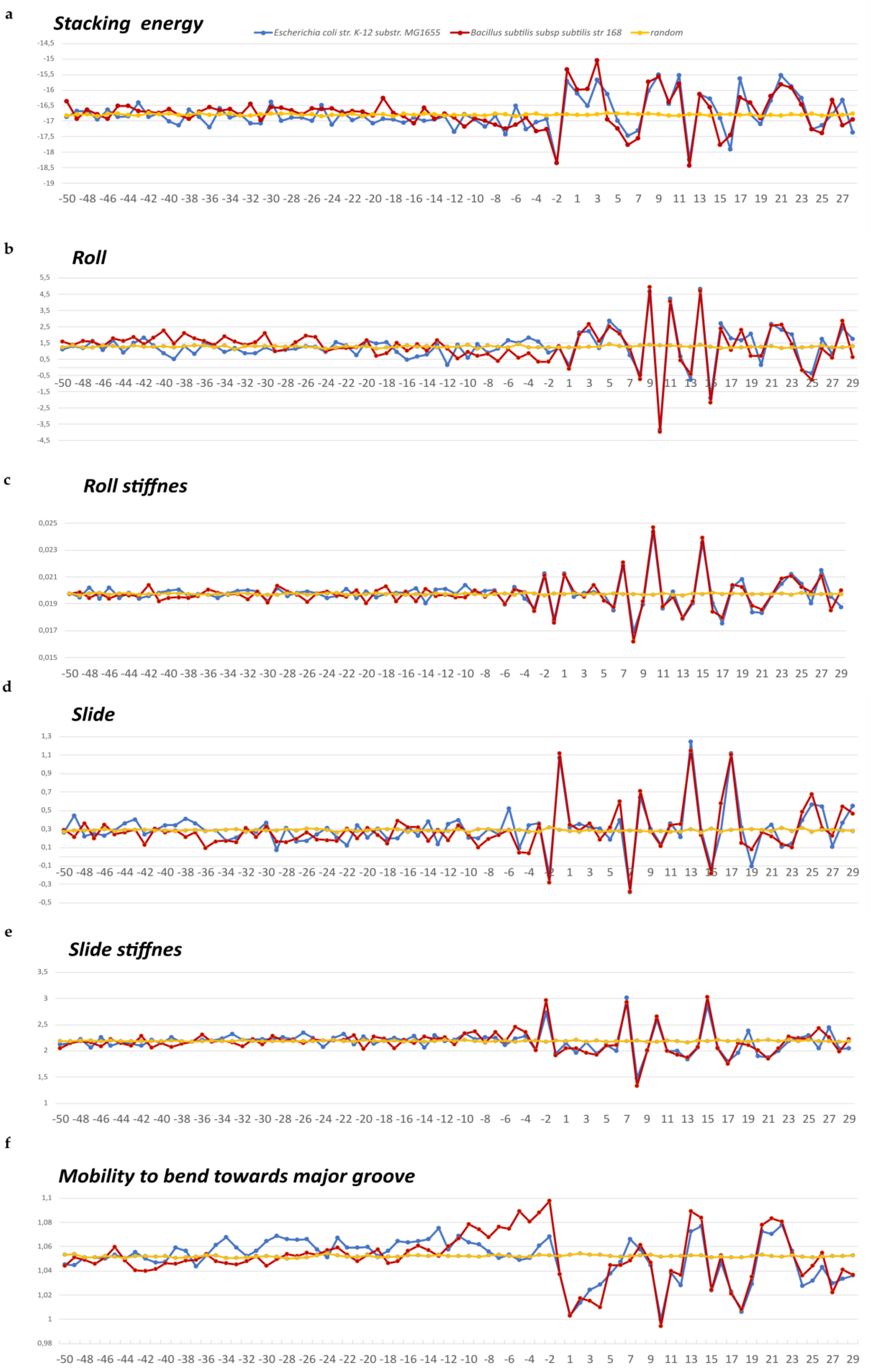
a
Stacking energy
Escherichia coli str. K-12 substr. MG1655
Bacillus subtilis subsp subtilis str 168
random
b
Roll
c
Roll stiffnes
d
Slide
e
Slide stiffnes
f
Mobility to bend towards major groove

Figure S1 (a–f): Local variations of the values of physical and structural parameters in core promoter regions of tRNA genes of E. coli (in blue) and B.subtilis (in red) along with the profile of the 80 bp set of 3000 computer-simulated random nucleotide sequences (in yellow). (**a**) Stacking energy (in kcal/mol). (**b**) Roll (in degrees). (**c**) Stiffness of the duplex structure to Roll alteration (in kcal/mol degree). (**d**) Slide (in angstroms). (**e**) Stiffness of the duplex structure to Slide alteration (in kcal/mol angstrom). (**f**) Mobility to bend towards major groove (in mobility units).

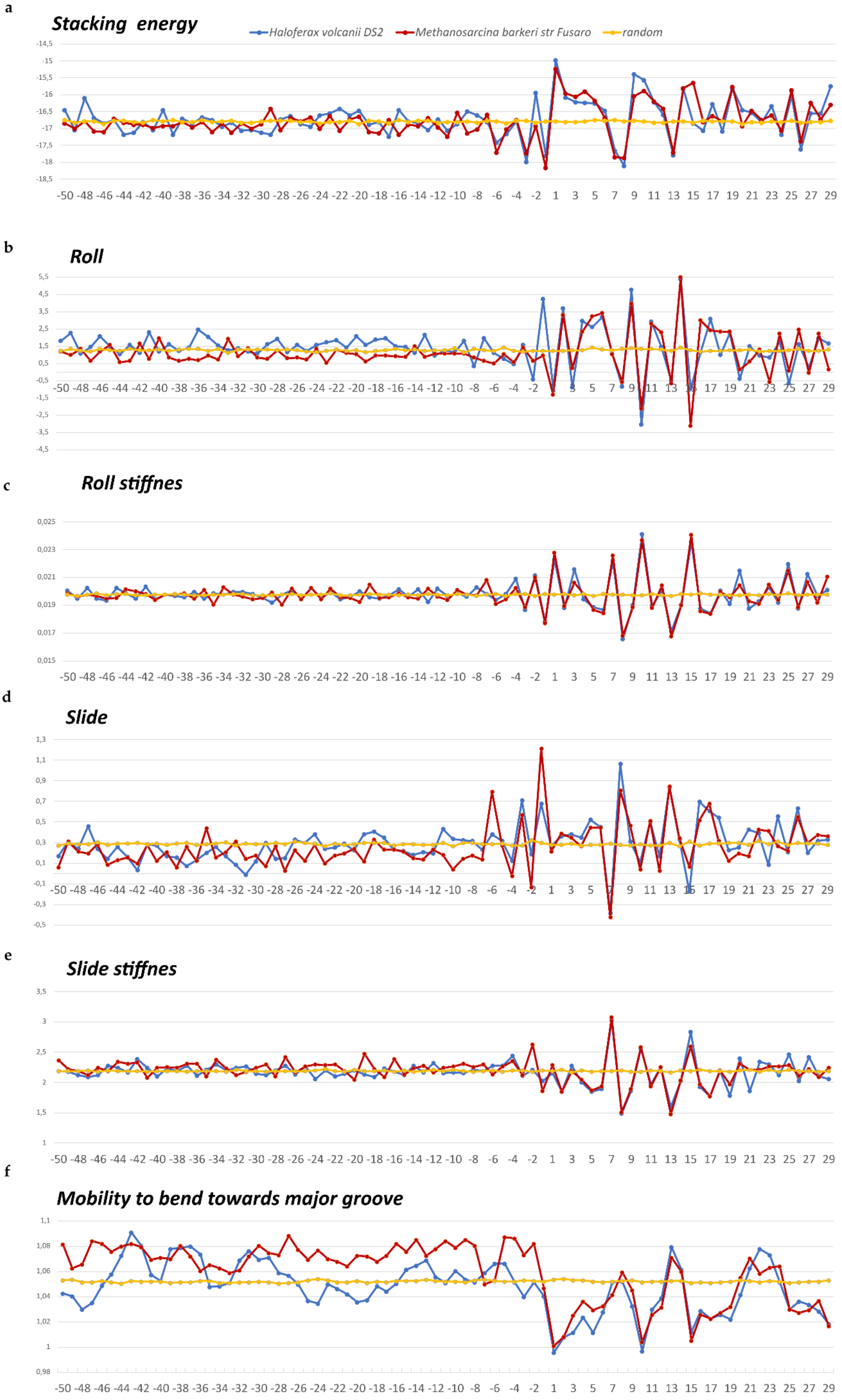
a
Stacking energy
Haloferax volcanii DS2
Methanosarcina barkeri str Fusaro
random
b
Roll
c
Roll stiffnes
d
Slide
e
Slide stiffnes
f
Mobility to bend towards major groove

Figure S2. Local variations of the values of physical and structural parameters in naked DNA in core promoter regions of mRNA genes, transcribed by Pol II, in genomes of M. barkeri (in red) and H. volcanii (in blue). (**a**) Stacking energy (in kcal/mol). (**b**) Roll (in degrees). (**c**) Stiffness of the duplex structure to Roll alteration (in kcal/mol degree). (**d**) Slide (in angstroms). (**e**) Stiffness of the duplex structure to Slide alteration (in kcal/mol angstrom). (**f**) Mobility to bend towards major groove (in mobility units).

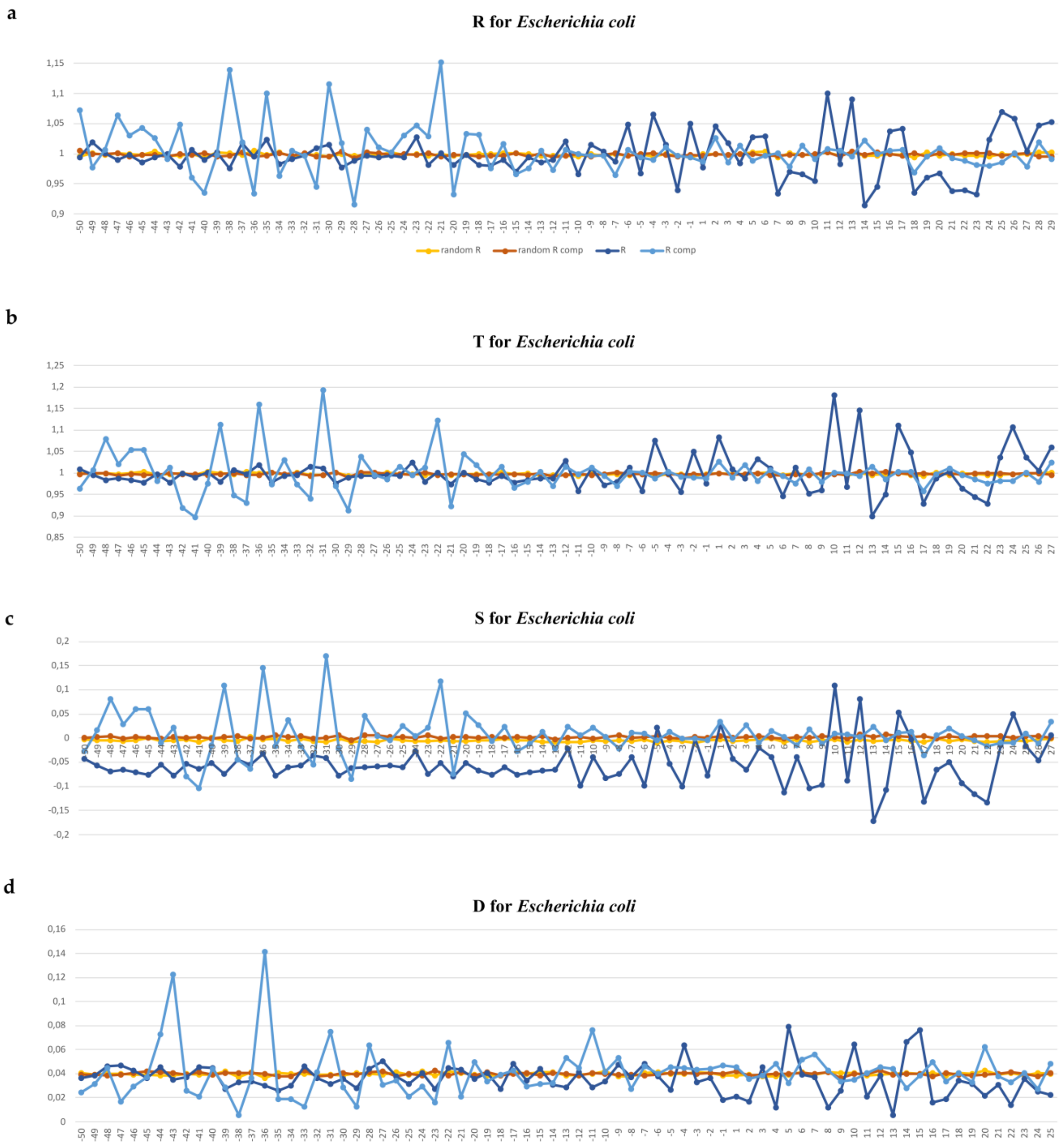

Figure S3 (a–d): Profiles of ultrasonic cleavage indexes and DNase I cleavage indexes for E. coli; (a) Profiles

of the relative intensities of ultrasonic cleavage of 16 dinucleotides (R); (b) profiles of the relative intensities of ultrasonic cleavage of 256 tetranucleotides (T); (c) profiles of indices S = (T − R)/R, (g) DNase I cleavage indices at hexanucleotide level of resolution (D); (d) DNase I cleavage indices at hexanucleotide level of resolution (D);

a

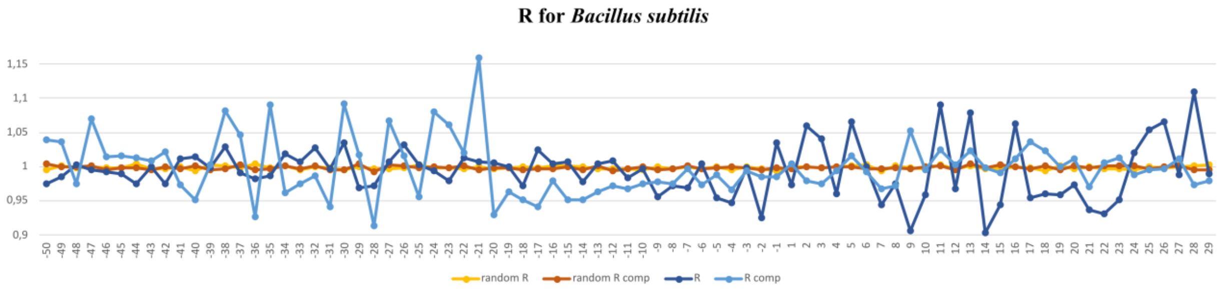

b

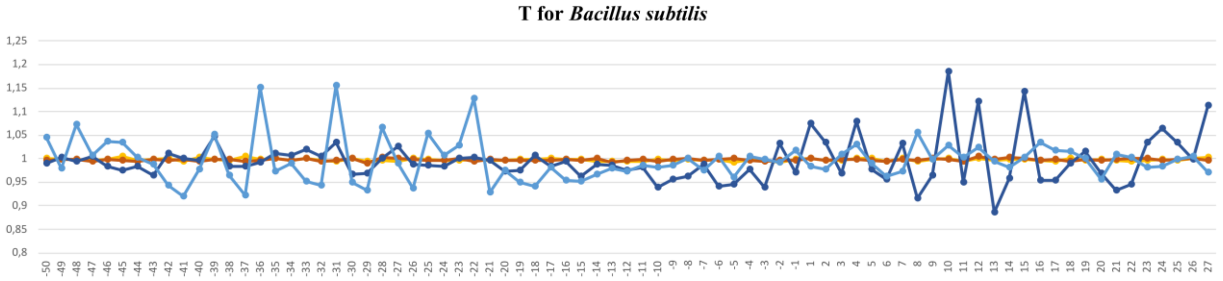

c

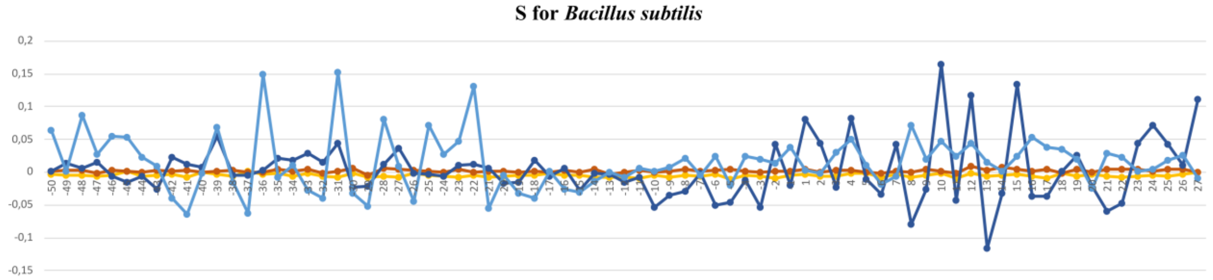

d

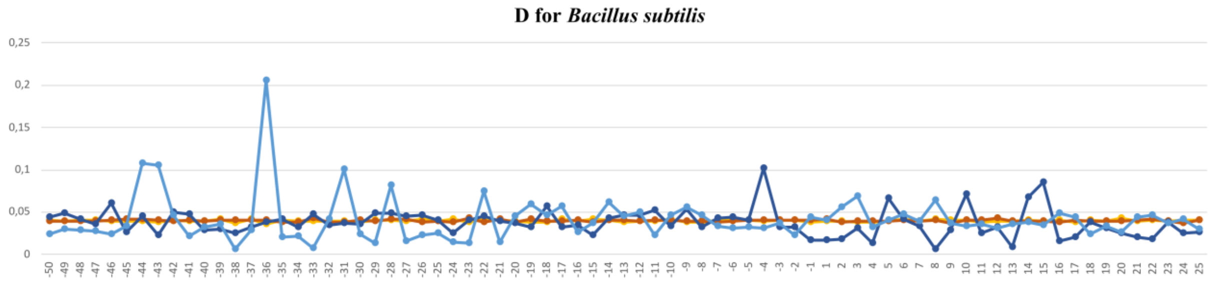

Figure S4 (a–d): Profiles of ultrasonic cleavage indexes and DNase I cleavage indexes for B. subtilis; (a) Profiles of the relative intensities of ultrasonic cleavage of 16 dinucleotides (R); (b) profiles of the

relative intensities of ultrasonic cleavage of 256 tetranucleotides (T); (c) profiles of indices S = (T − R)/R, (g) DNase I cleavage indices at hexanucleotide level of resolution (D); (d) DNase I cleavage indices at hexanucleotide level of resolution (D);

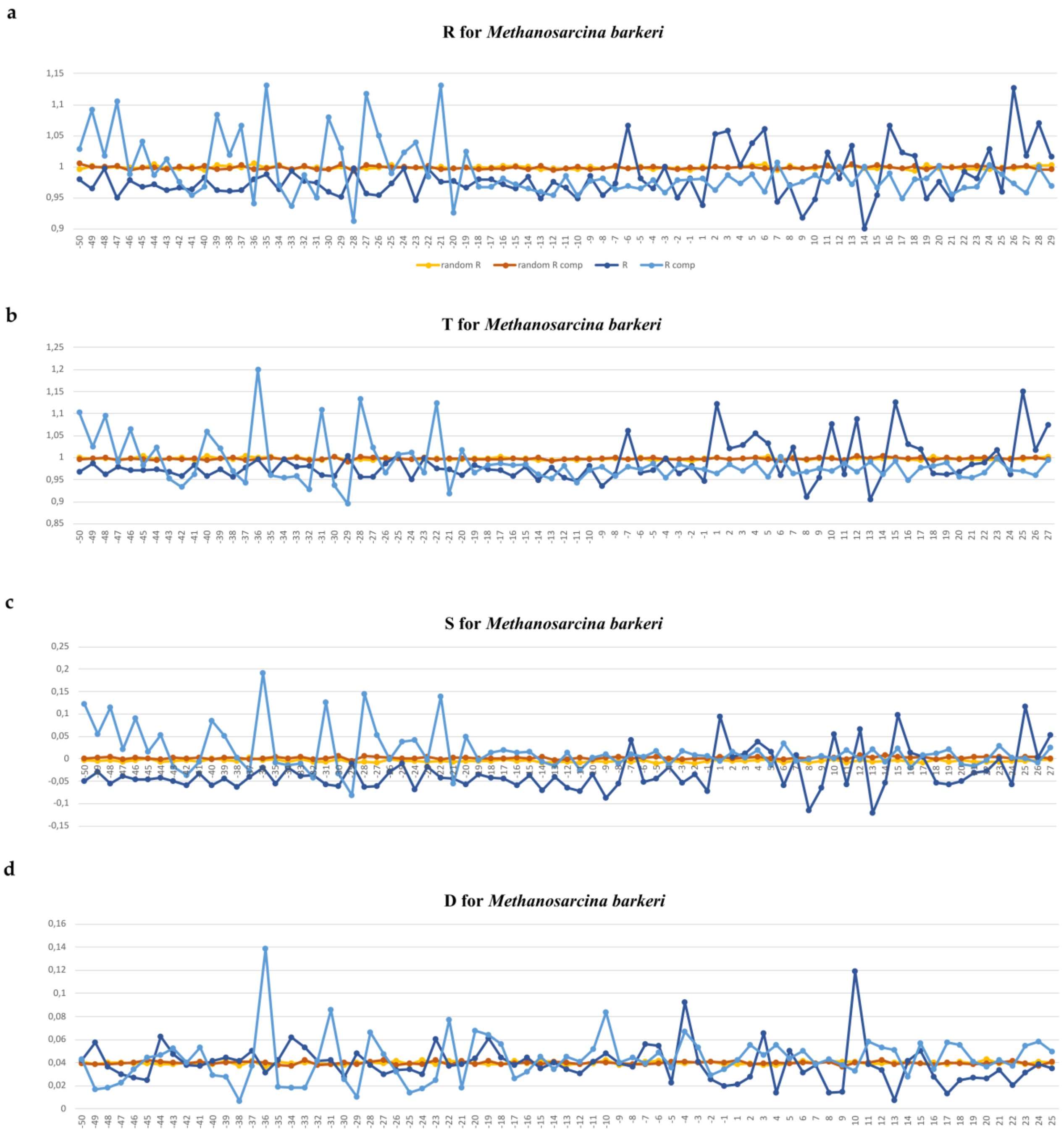

Figure S5 (a–d): Profiles of ultrasonic cleavage indexes and DNase I cleavage indexes for M. barkeri; (a) Profiles of the relative intensities of ultrasonic cleavage of 16 dinucleotides (R); (b) profiles of the relative intensities of ultrasonic cleavage of 256 tetranucleotides (T); (c) profiles of indices S = (T − R)/R, (g) DNase I cleavage indices at hexanucleotide level of resolution (D); (d) DNase I cleavage indices at hexanucleotide level of resolution (D);

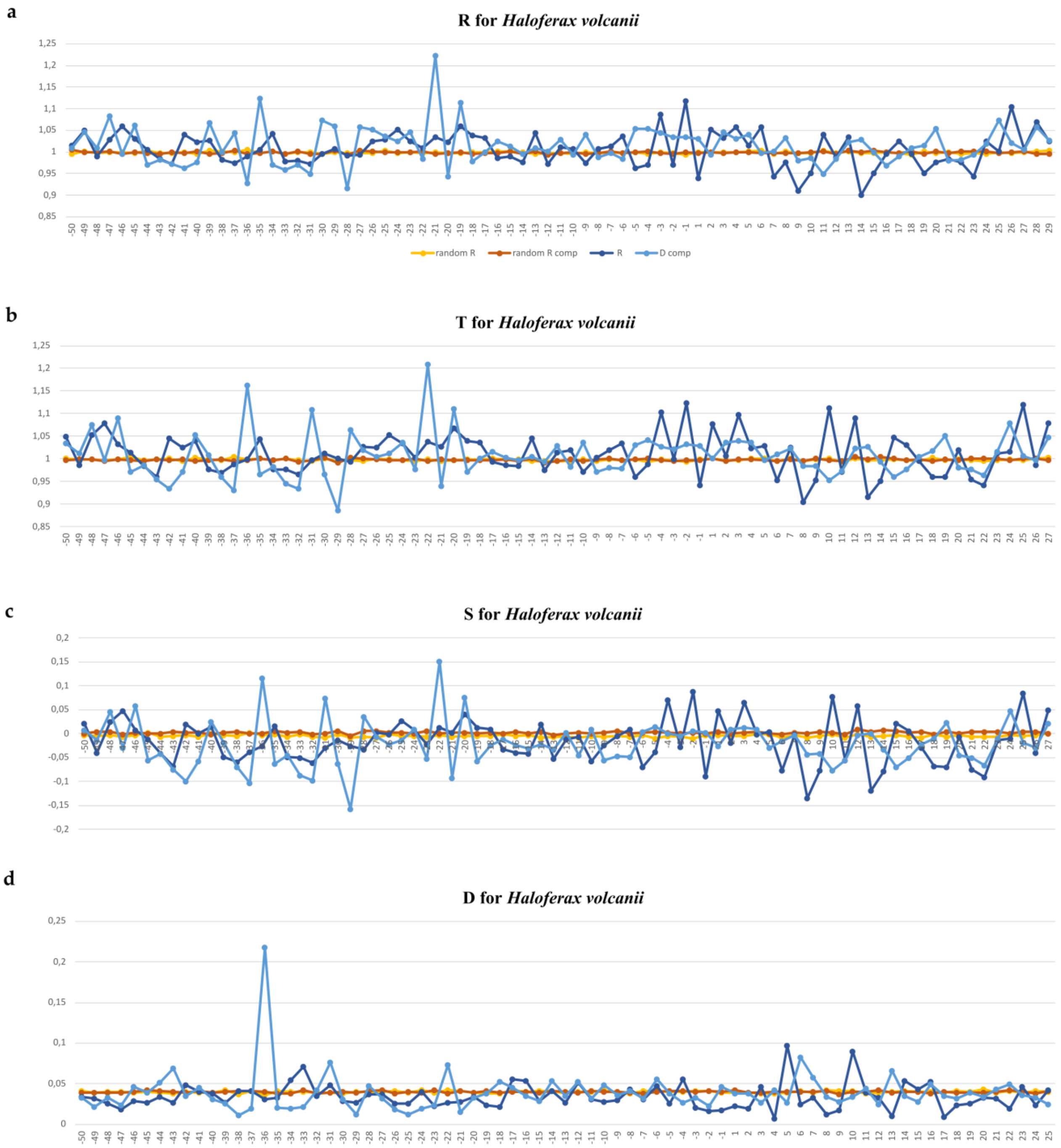

Figure S6 (a–d): Profiles of ultrasonic cleavage indexes and DNase I cleavage indexes for H. volcanii; (a) Profiles of the relative intensities of ultrasonic cleavage of 16 dinucleotides (R); (b) profiles of the relative intensities of ultrasonic cleavage of 256 tetranucleotides (T); (c) profiles of indices S = (T − R)/R, (g) DNase I cleavage indices at hexanucleotide level of resolution (D); (d) DNase I cleavage indices at hexanucleotide level of resolution (D);

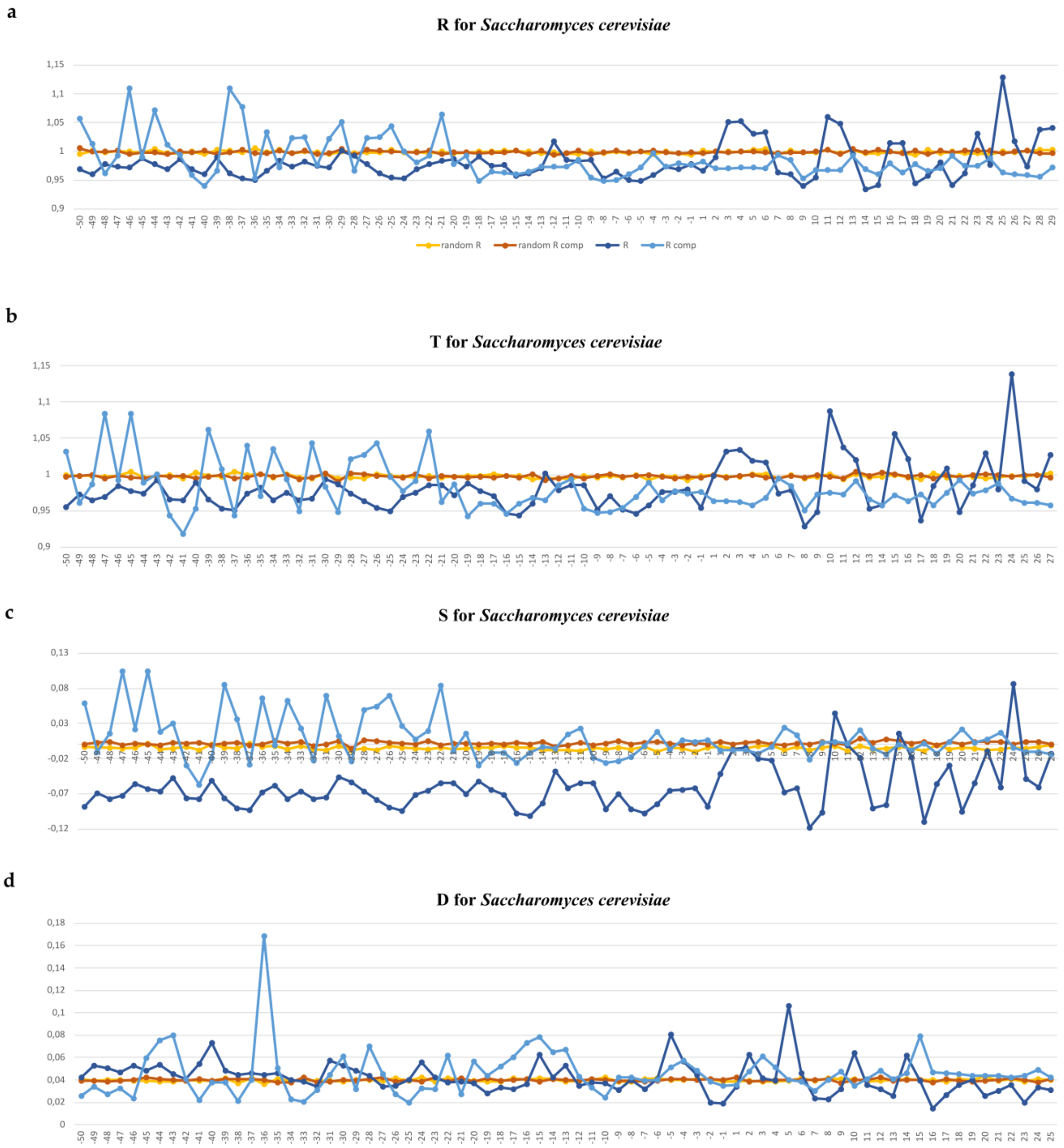

Figure S7 (a–d): Profiles of ultrasonic cleavage indexes and DNase I cleavage indexes for S. Cerevisiae; (a) Profiles of the relative intensities of ultrasonic cleavage of 16 dinucleotides (R); (b) profiles of the relative intensities of ultrasonic cleavage of 256 tetranucleotides (T); (c) profiles of indices S = (T − R)/R, (g) DNase I cleavage indices at hexanucleotide level of resolution (D); (d) DNase I cleavage indices at hexanucleotide level of resolution (D);

a

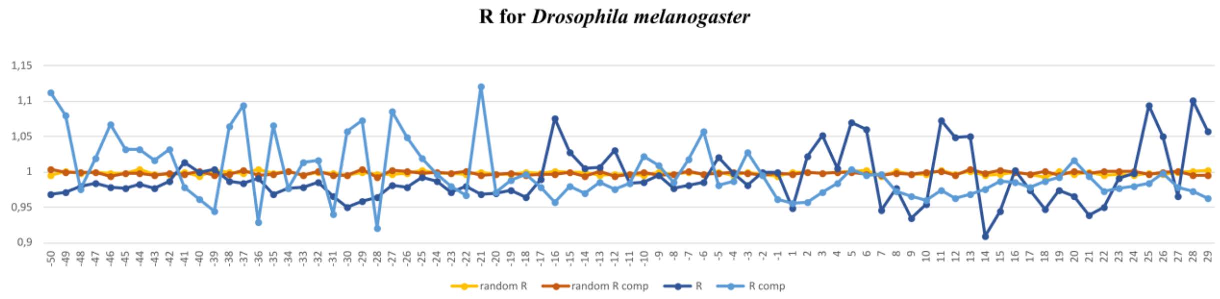

b

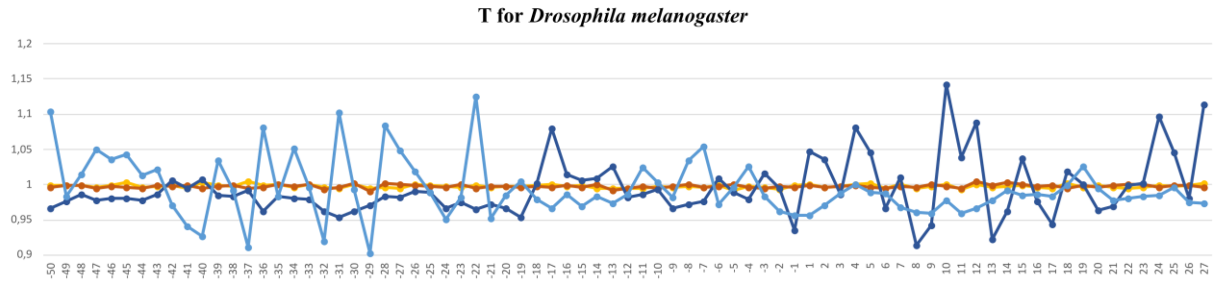

c

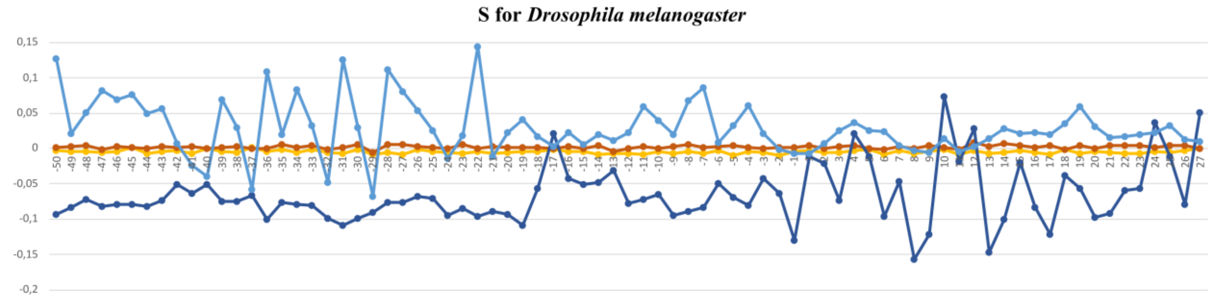

d

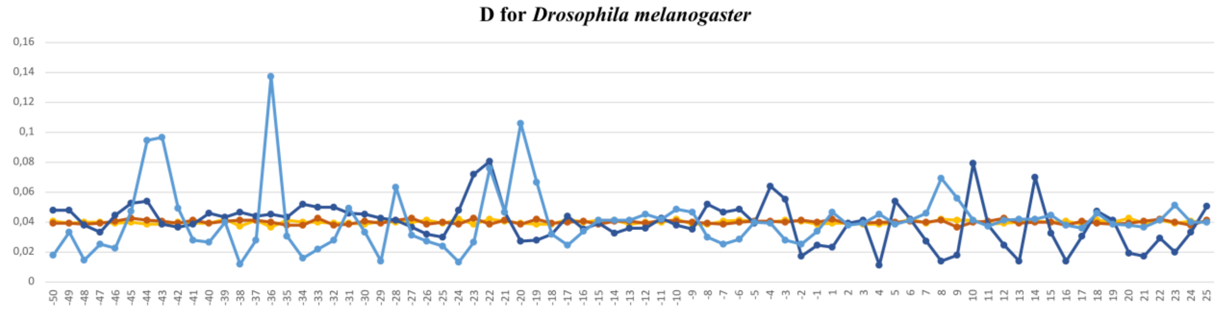

Figure S8 (a–d): Profiles of ultrasonic cleavage indexes and DNase I cleavage indexes for D. Melanogaster;(a) Profiles of the relative intensities of ultrasonic cleavage of 16 dinucleotides (R); (b) profiles of the relative intensities of ultrasonic cleavage of 256 tetranucleotides (T); (c) profiles of indices S = (T − R)/R, (g) DNase I cleavage indices at hexanucleotide level of resolution (D); (d) DNase I cleavage indices at hexanucleotide level of resolution (D);

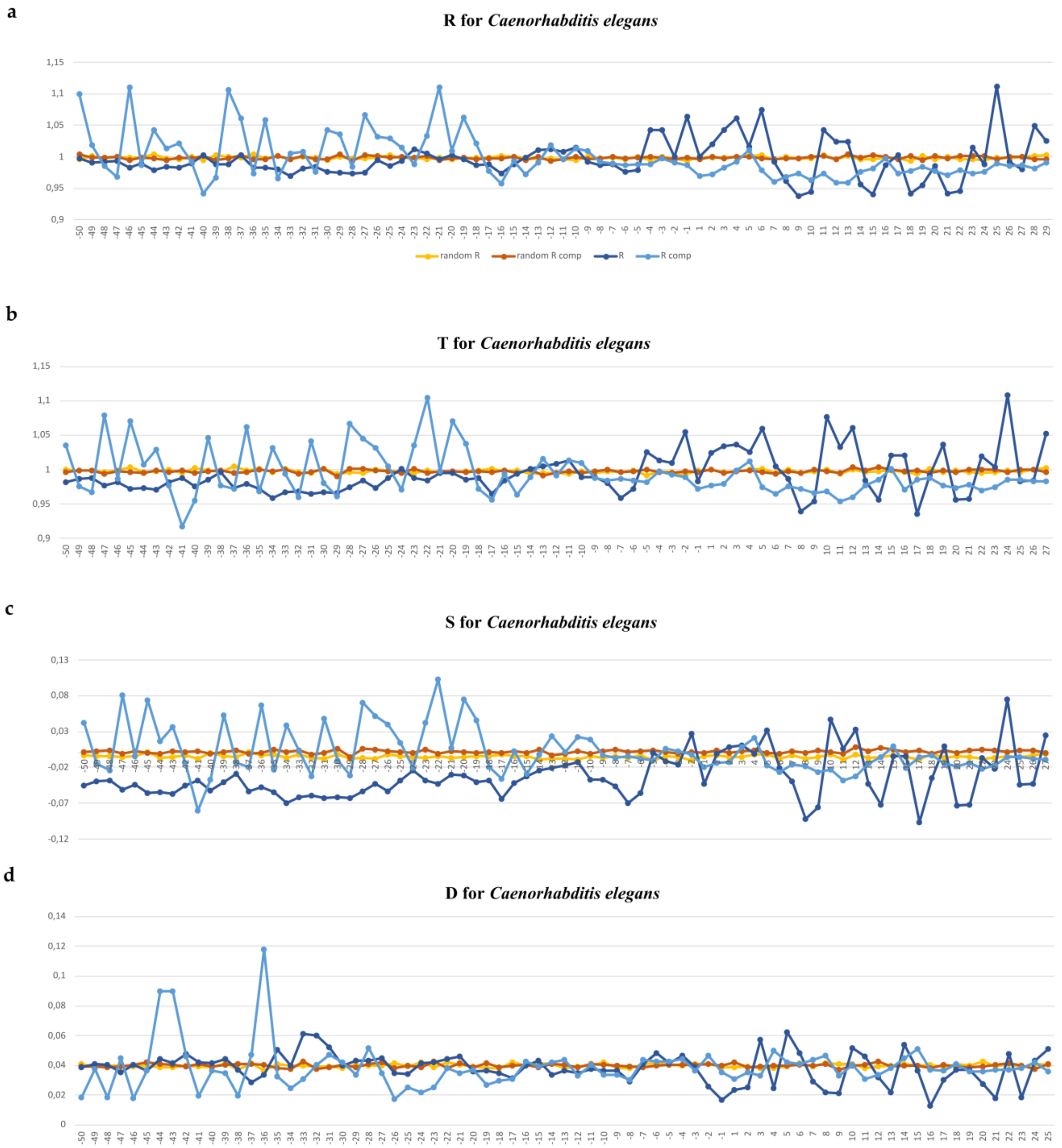

Figure S9 (a–d): Profiles of ultrasonic cleavage indexes and DNase I cleavage indexes for C. elegans; (a) Profiles of the relative intensities of ultrasonic cleavage of 16 dinucleotides (R); (b) profiles of the relative intensities of ultrasonic cleavage of 256 tetranucleotides (T); (c) profiles of indices S = (T − R)/R, (g) DNase I cleavage indices at hexanucleotide level of resolution (D); (d) DNase I cleavage indices at hexanucleotide level of resolution (D);

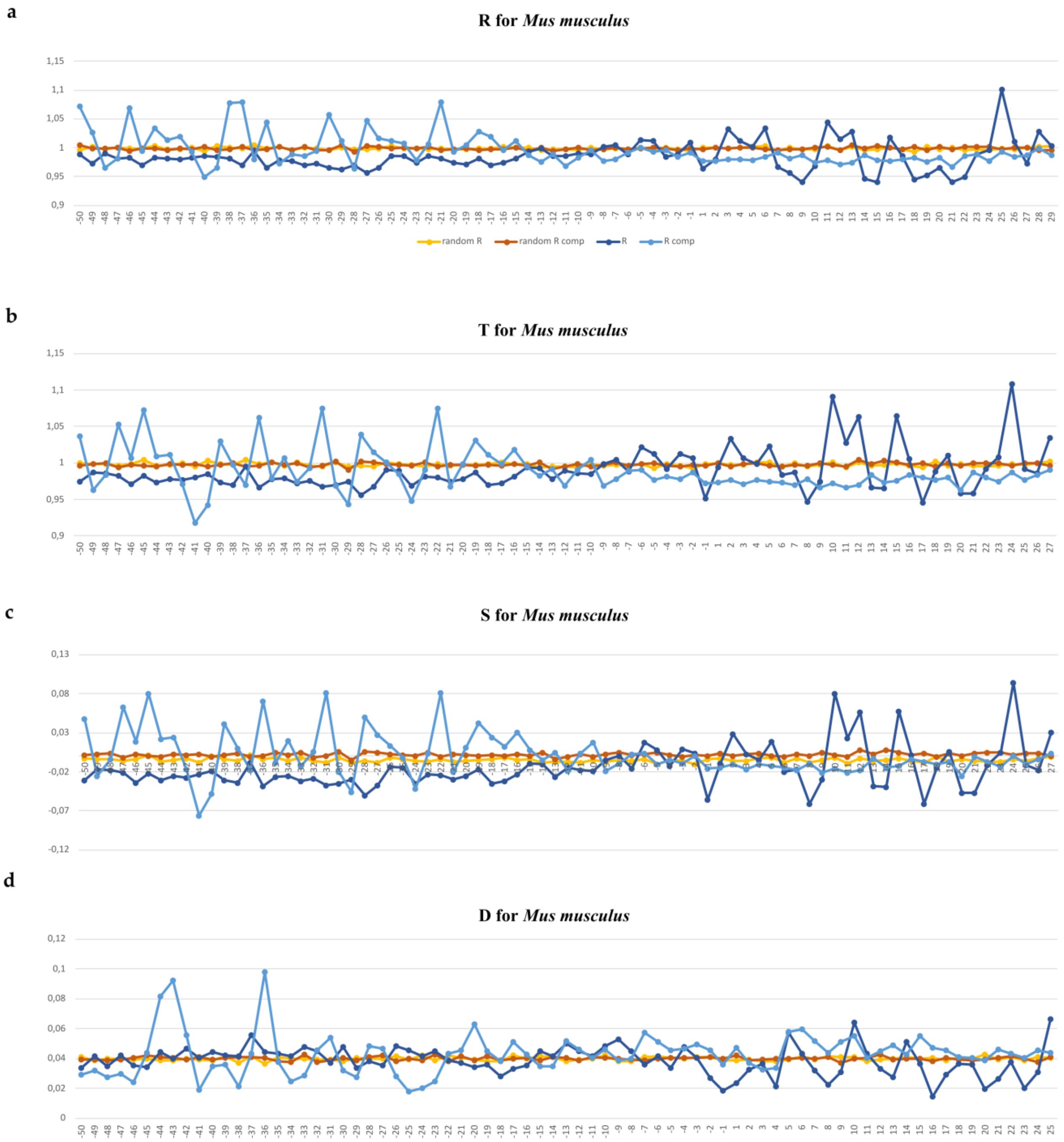

Figure S10 (a–d): Profiles of ultrasonic cleavage indexes and DNase I cleavage indexes for M. Musculus; (a) Profiles of the relative intensities of ultrasonic cleavage of 16 dinucleotides (R); (b) profiles of the relative intensities of ultrasonic cleavage of 256 tetranucleotides (T); (c) profiles of indices S = (T − R)/R, (g) DNase I cleavage indices at hexanucleotide level of resolution (D); (d) DNase I cleavage indices at hexanucleotide level of resolution (D);

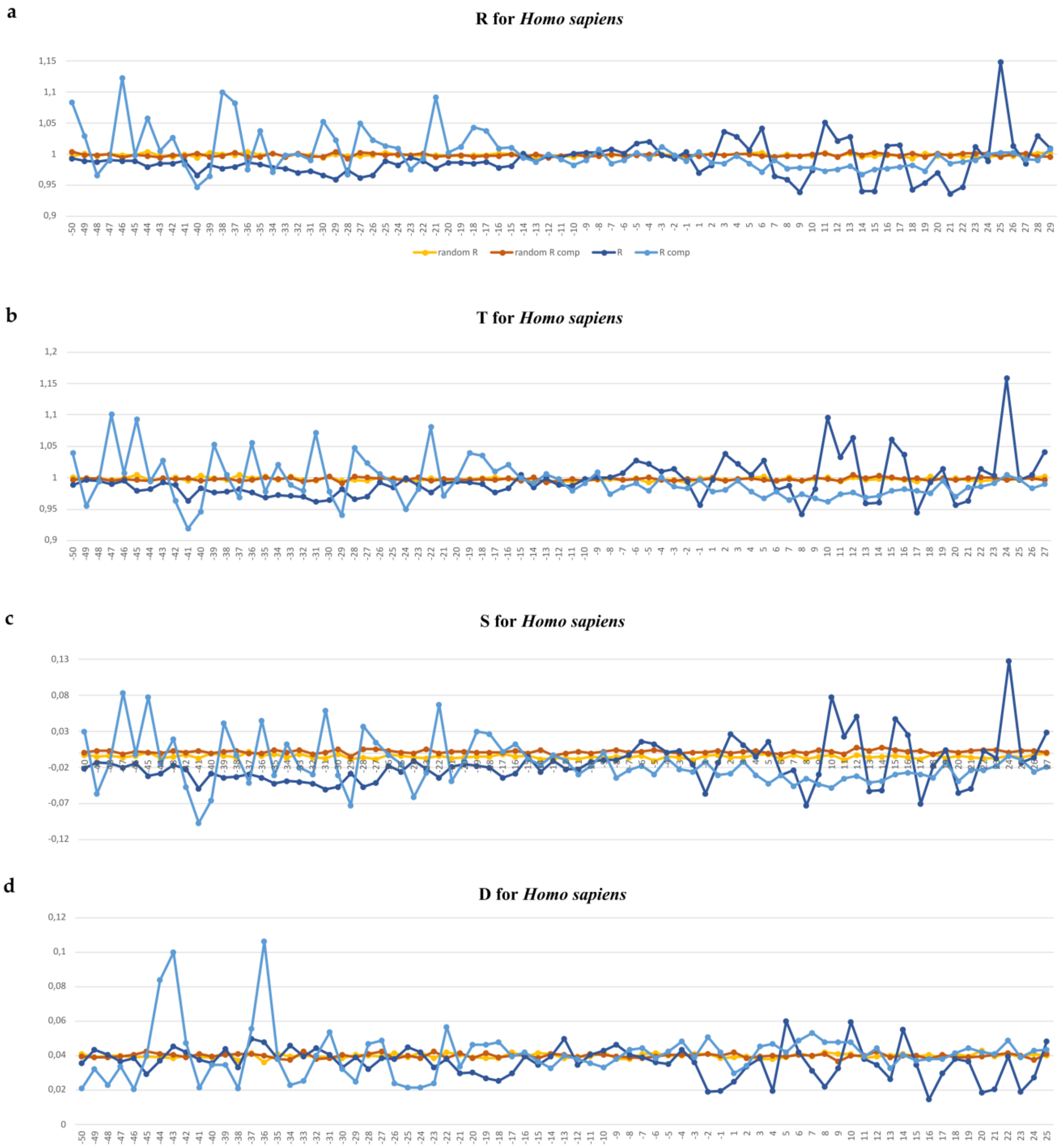

Figure S11 (a–d): Profiles of ultrasonic cleavage indexes and DNase I cleavage indexes for H. sapiens; (a) Profiles of the relative intensities of ultrasonic cleavage of 16 dinucleotides (R); (b) profiles of the relative intensities of ultrasonic cleavage of 256 tetranucleotides (T); (c) profiles of indices S = (T − R)/R, (g) DNase I cleavage indices at hexanucleotide level of resolution (D); (d) DNase I cleavage indices at hexanucleotide level of resolution (D);

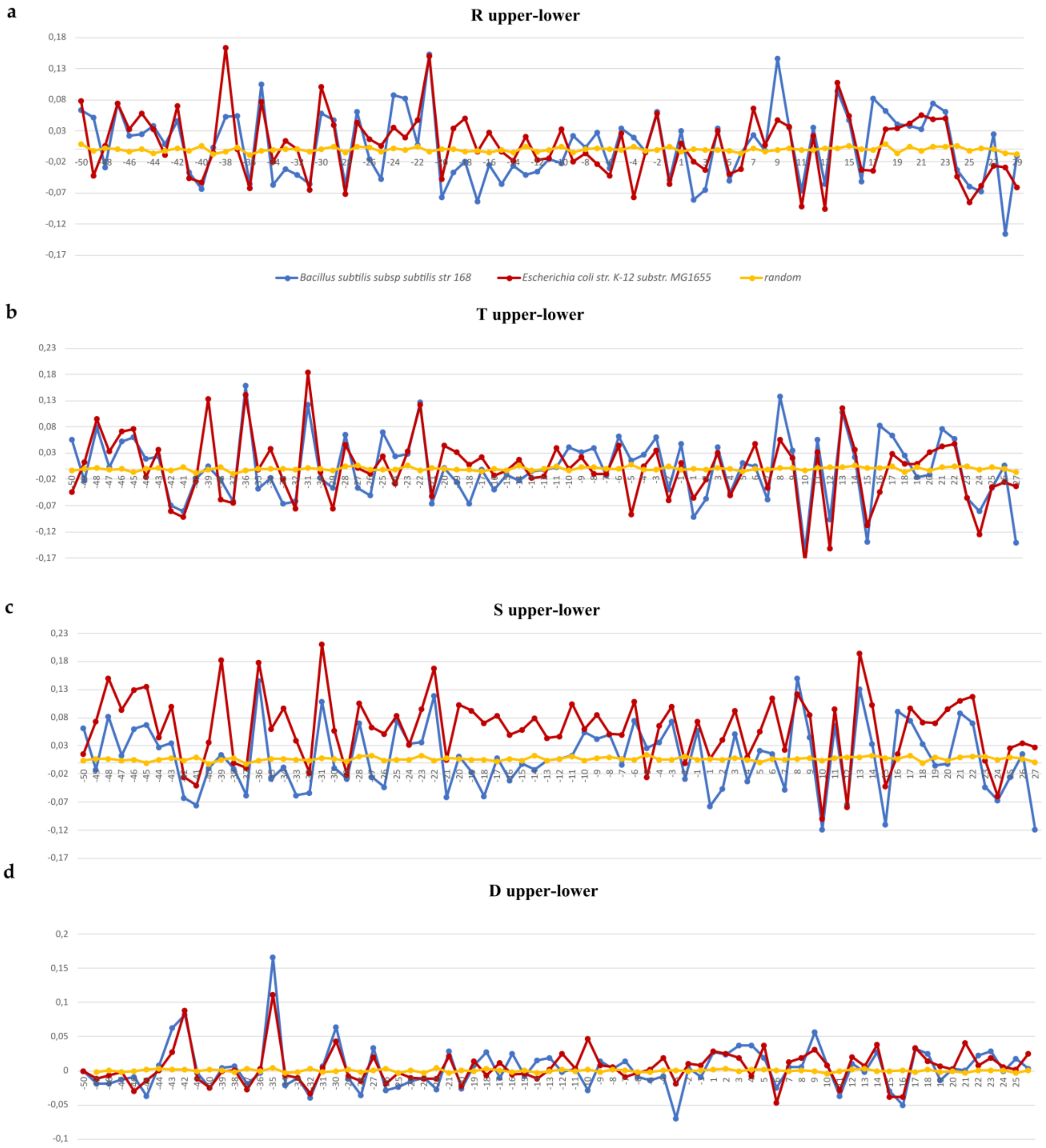

Figure S12 (a–d): Profiles of the differences of cleavage intensities between the complementary chains in E. coli (in red) and B. subtilis (in blue), random 3000 sequences (in yellow); (a) Profiles of the relative intensities of ultrasonic cleavage of 16 dinucleotides (R); (b) profiles of the relative intensities of ultrasonic cleavage of 256 tetranucleotides (T); (c) profiles of indices S = (T − R)/R, (g) DNase I cleavage indices at hexanucleotide level of resolution (D); (d) DNase I cleavage indices at hexanucleotide level of resolution (D);

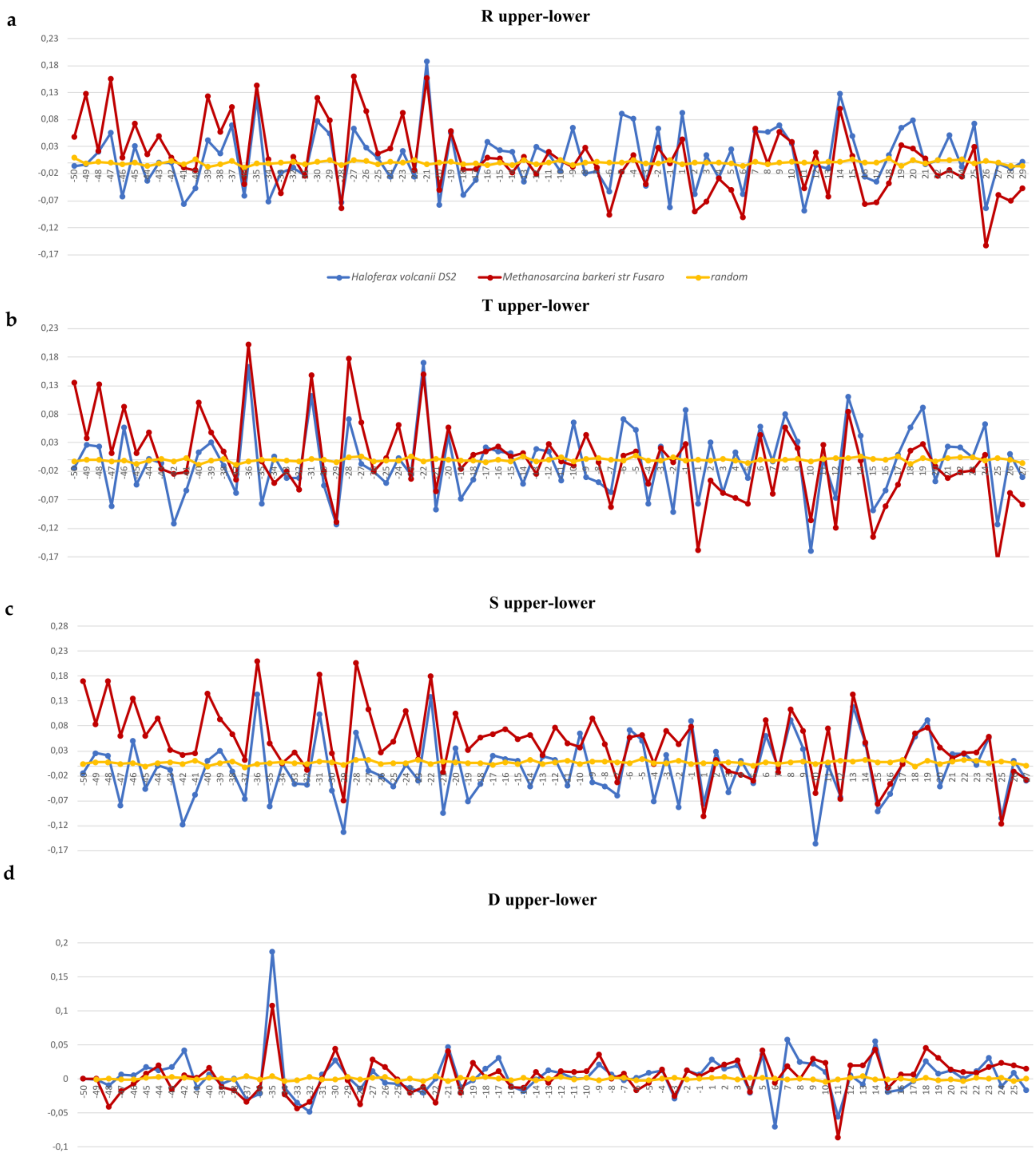

Figure S13 (a–d): Profiles of the differences of cleavage intensities between the complementary chains in M. barkeri (in red) and H. volcanii (in blue), random 3000 sequences (in yellow); (a) Profiles of the relative intensities of ultrasonic cleavage of 16 dinucleotides (R); (b) profiles of the relative intensities of ultrasonic cleavage of 256 tetranucleotides (T); (c) profiles of indices S = (T − R)/R, (g) DNase I cleavage indices at hexanucleotide level of resolution (D); (d) DNase I cleavage indices at hexanucleotide level of resolution (D);

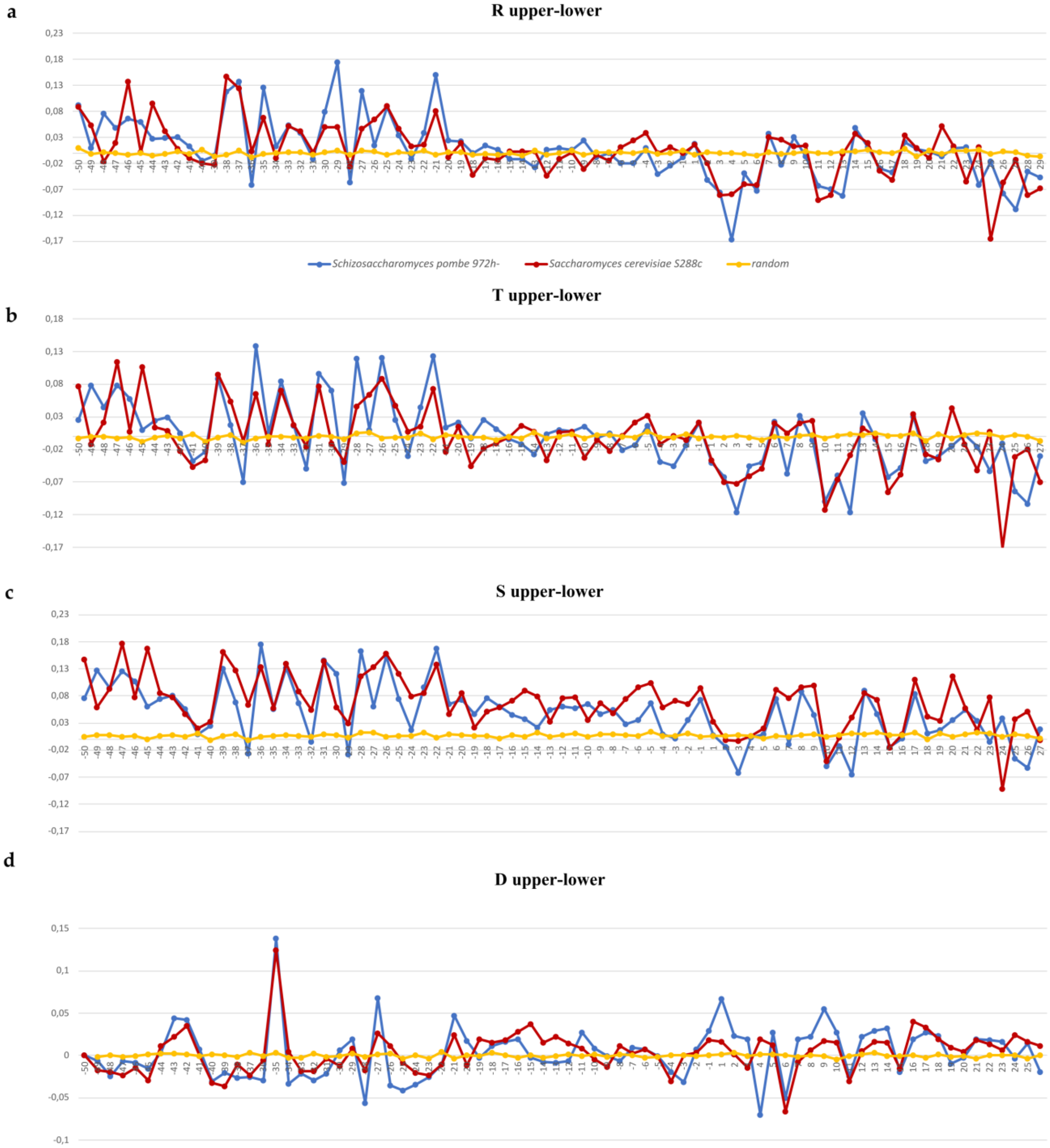

Figure S14 (a–d): Profiles of the differences of cleavage intensities between the complementary chains in S. pombe (in blue) and S. cerevisiae (in red), random 3000 sequences (in yellow); (a) Profiles of the relative intensities of ultrasonic cleavage of 16 dinucleotides (R); (b) profiles of the relative intensities of ultrasonic cleavage of 256 tetranucleotides (T); (c) profiles of indices S = (T − R)/R, (g) DNase I cleavage indices at hexanucleotide level of resolution (D); (d) DNase I cleavage indices at hexanucleotide level of resolution (D);

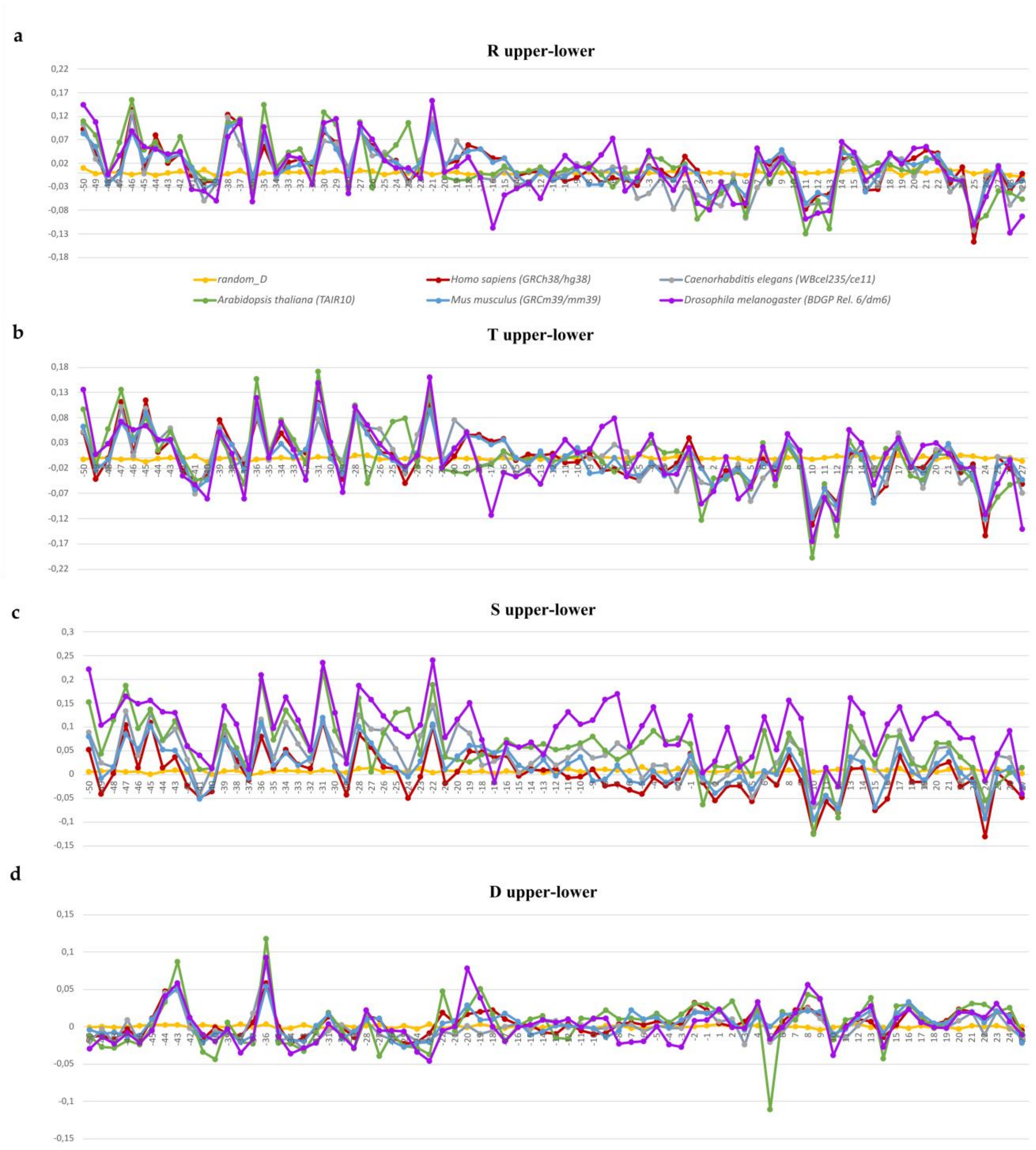

Figure S15 (a–d): Profiles of the differences of cleavage intensities between the complementary chains in A. thaliana (in green), D. melanogaster (in purple), C. elegans (in grey), M. musculus (in blue) and H. sapiens (in red) ,random 3000 sequences (in yellow); ; (a) Profiles of the relative intensities of ultrasonic cleavage of 16 dinucleotides (R); (b) profiles of the relative intensities of ultrasonic cleavage of 256 tetranucleotides (T); (c) profiles of indices S = (T − R)/R, (g) DNase I cleavage indices at hexanucleotide level of resolution (D); (d) DNase I cleavage indices at hexanucleotide level of resolution (D);
